# FHbp variants among meningococci of serogroup B in Italy: Evolution and selective pressure, 2014–2017

**DOI:** 10.1371/journal.pone.0277976

**Published:** 2023-02-16

**Authors:** Alessandra Lo Presti, Anna Carannante, Cecilia Fazio, Arianna Neri, Paola Vacca, Luigina Ambrosio, Florigio Lista, Silvia Fillo, Paola Stefanelli

**Affiliations:** 1 Department of Infectious Diseases, Istituto Superiore di Sanità, Rome, Italy; 2 Scientific Department, Army Medical Center, Rome, Italy; University of Auckland, NEW ZEALAND

## Abstract

**Background:**

*Neisseria meningitidis* (meningococcus) is the causative agent of invasive meningococcal disease (IMD). Meningococcus of serogroup B (MenB) is one of the main serogroup causing IMD. MenB strains may be prevented by meningococcal B vaccines. In particular, vaccines with Factor H-binding protein (FHbp), classified into two subfamilies (A or B) or in three variants (v1, v2 or v3), are those available. The objective of the study was to investigate the phylogenetic relationships of FHbp subfamilies A and B (variants v1, v2 or v3) genes and proteins, together with their evolution patterns and selective pressure.

**Materials and methods:**

Overall, alignments of FHbp nucleotide and protein sequence from 155 MenB samples collected in different parts of Italy, from 2014 to 2017, were analyzed by ClustalW. JModeltest and the Smart Model Selection software were used for the statistical selection of the best-fit substitution models for nucleotide and protein alignments. Site-specific positive and negative selection were estimated through the HYPHY package. The phylogenetic signal was investigated with the likelihood mapping method. The Maximum Likelihood (ML) phylogenetic reconstructions were performed with Phyml.

**Results:**

The phylogenic analysis identified different clusters within the FHbp subfamily A and B variants, confirming sequence diversity. The pattern of selective pressure in our study indicated that subfamily B FHbp sequences are subjected to greater variations and positive selective pressure respect to subfamily A, with 16 positively supported selected sites identified.

**Conclusion:**

The study pointed out the need for continued genomic surveillance for meningococci to monitor selective pressure and amino acidic changes. Monitoring the genetic diversity and molecular evolution of FHbp variants may be useful to investigate genetic diversity which may emerge over time.

## Introduction

*Neisseria meningitidis* is the causative agent of invasive meningococcal disease (IMD), predominantly presenting as meningitis and/or septicemia.

*N*. *meningitidis* is classified into 12 serogroups with six (A, B, C, W, X and Y) responsible for the majority of IMD cases worldwide and an average notification rate of around 0.6/100,000 people in European countries [1–4].

In Italy, since 2015 the incidence has fluctuated around the value of 0.3 cases/100,000 inhabitants. In 2020 the rate decreased to 0.12 cases/100,000 inhabitants, as a consequence of the impact of restrictive measures adopted to mitigate the SARS-CoV-2 pandemic [5].

MenB may be prevented by the available meningococcal B vaccines targeted with the Factor H-binding protein (FHbp). The FHbp, also known as GNA1870 (Genome-Derived Neisseria Antigen 1870) or LP2086 (lipoprotein LP2086) [6, 7], is one of the components of 4CMenB vaccine [8, 9] and the single target of the bivalent MenB vaccine [10, 11]. The FHbp binds specifically to human complement regulatory protein factor H and inhibits the alternative complement pathway [12], thus improving the survival of *N*. *meningitidis* in blood.

From an evolutionary point of view, FHbp can be classified into two subfamilies (A or B) or three variants (v1, v2 or v3). Recombination at FHbp contributes to the antigenic diversity within some clonal complex of MenB isolates [6, 10].

As reported in our previous study [13], based on a panel of MenB strains causing IMD in Italy from 2014 to 2017, the A05 variant resulted as predominant (n = 14; 19.7%), followed by A06 (n = 10; 14.1%) and A22 (n = 10; 14.1%); the B231 variant accounted for 32.5%, B03 for 13.2% and B24 for 10.8% [13]. In particular, the A05 and B231 were the most frequent variants associated with the clonal complexes cc213 and cc162, respectively [13]. All MenB with the FHbp A05 variant displayed the PorA P1.22, 14 and 85.7% of them the FetA F5-5. The A05 variant was the only identified among meningococci belonging to cc213 [13]. The majority of MenB with the FHbp B231 variant showed the PorA P1.22, 14 (65.4%) and 84.6%, the FetA F3-6 [13].

The aims of this study were: i) to investigate the relationships between FHbp subfamilies A and B (variants v1, v2, v3) performing the phylogenetic analysis of *fhbp* genes and proteins; ii) to analyze and compare the patterns of sequence evolution and the selective pressure on FHbp subfamily A and B sequences.

## Materials and methods

### Phylogenetic analysis

The FHbp nucleotide and protein sequence alignments were performed with ClustalW [14] on 155 MenB collected from 2014 to 2017, representative of the total IMD reported within the National Surveillance System (174 MenB samples sent to ISS, of which 109 were culture positive, 46 culture negative and 19 unsuitable for molecular analysis), and previously molecular characterized within the frame of a project, the results of which were already described [13]. For the 109 culture positive MenB samples the genomes were submitted into PubMLST.org database (http://pubmlst.org/neisseria/), S1 Table, and the multilocus sequence typing (MLST), PorA, FetA and FHbp typing were identified using whole genome sequencing (WGS).

Manual editing was performed by Bioedit [15]. JModeltest and Smart Model Selection software [16, 17] have been used for statistical selection of the best-fit substitution models for nucleotide and protein alignments, respectively. The phylogenetic signal in a data set of aligned DNA or amino acid sequences can be investigated with the likelihood mapping method by analyzing groups of four randomly chosen sequences, called quartets [18]. A quartet has three possible un-rooted tree topologies. The likelihood of each topology is estimated with the maximum likelihood method and the three likelihoods are reported as a dot in an equilateral triangle (the likelihood map). Three main areas can be distinguished in the map: the three corners representing fully resolved tree topologies (*i*.*e*., the presence of a treelike phylogenetic signal in the data), the center (which represents a star-like phylogeny), and the three areas on the sides that indicate a network-like phylogeny (*i*.*e*. the presence of recombination or conflicting phylogenetic signals). A substantial star-like signal (*i*.*e*. a star-like outburst of multiple phylogenetic lineages) is indicated by >33% dots falling within the central area, as confirmed by extensive simulation studies. Likelihood mapping analyses have been performed with the TREE-PUZZLE program by analyzing 10.000 random quartets [18], as already described [19, 20].

The Maximum Likelihood (ML) phylogenetic reconstructions were carried out with Phyml [21], by using the GTR + I + G and the JTT + G models, previously selected, respectively for the nucleotide and protein alignments. The statistical significance in the tree, has been evaluated by the bootstrap test (statistically supported values > 75%). The trees were visualized and exported using FigTree v. 1.4.4 (http://tree.bio.ed.ac.uk/software/figtree/).

### Selective pressure analysis

Site-specific positive and negative selection were estimated through the HYPHY package which has been designed to provide a flexible and unified platform for carrying out likelihood-based analyses on multiple alignments of molecular sequence data, with the emphasis on studies of patterns of sequence evolution [22].

Selective pressure analysis has been conducted separately on the FHbp subfamily A and B sequences (or v1, v2 or v3), in order to compare the pattern and sites of evolution between them. Two different algorithms were used: the Fixed-effects likelihood (FEL), which fits an ω rate to every site and uses the likelihood ratio to test if dN ≠ dS; and the Random—effects likelihood (REL), a variant of the Nielsen–Yang approach [23], which assumes that a discrete distribution of rates exists across sites and allows both dS and dN to vary independently site by site. These methods have been described in detail elsewhere [22]. In order to select sites under selective pressure and keep our test conservative, a p value of ≤ 0.1 or a posterior probability of ≥ 0.9 as relaxed critical values [22, 24] was assumed. Part of the analysis has been also conducted by using the web-server Datamonkey [24, 25]. For the evolutionary analysis, the reference sequence (Uniprot Identifier: C6KHT4) was used to trace the exact position of the amino acids found under positive or negative selection.

## Results

### Phylogenetic analysis

**Fig 1:**
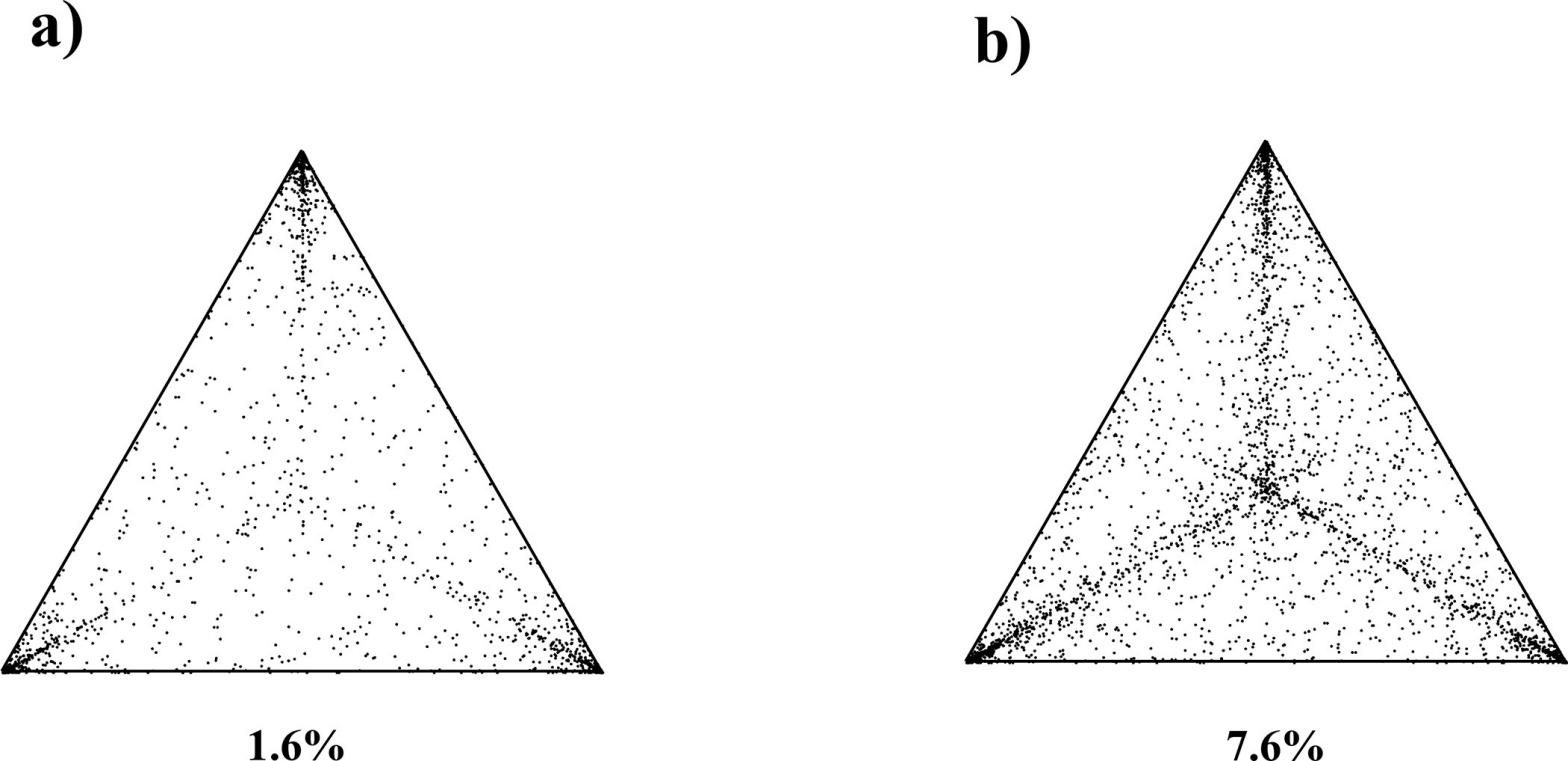
Likelihood mapping analysis of serogroup B *Neisseria meningitidis* FHbp nucleotide (panel a) and protein (panel b) alignments. Numbers indicate the percentage of dots in the centre of the triangle corresponding to phylogenetic noise (star-like trees).

Fig 1 shows the likelihood mapping analysis for FHbp nucleotide (panel a) and protein (panel b) alignments. Evaluation of 10,000 random quartets showed that 95.6% and 86.4% of the randomly chosen quartets, respectively for nucleotide and protein alignments were distributed in the corners of the likelihood map (representing the tree-like signal) and only 1.6% and 7.6% (respectively for the nucleotide and protein alignments) were concentrated in the center indicating low “*noise*”—“*star-like signal*” and well resolved trees topologies.

**Fig 2:**
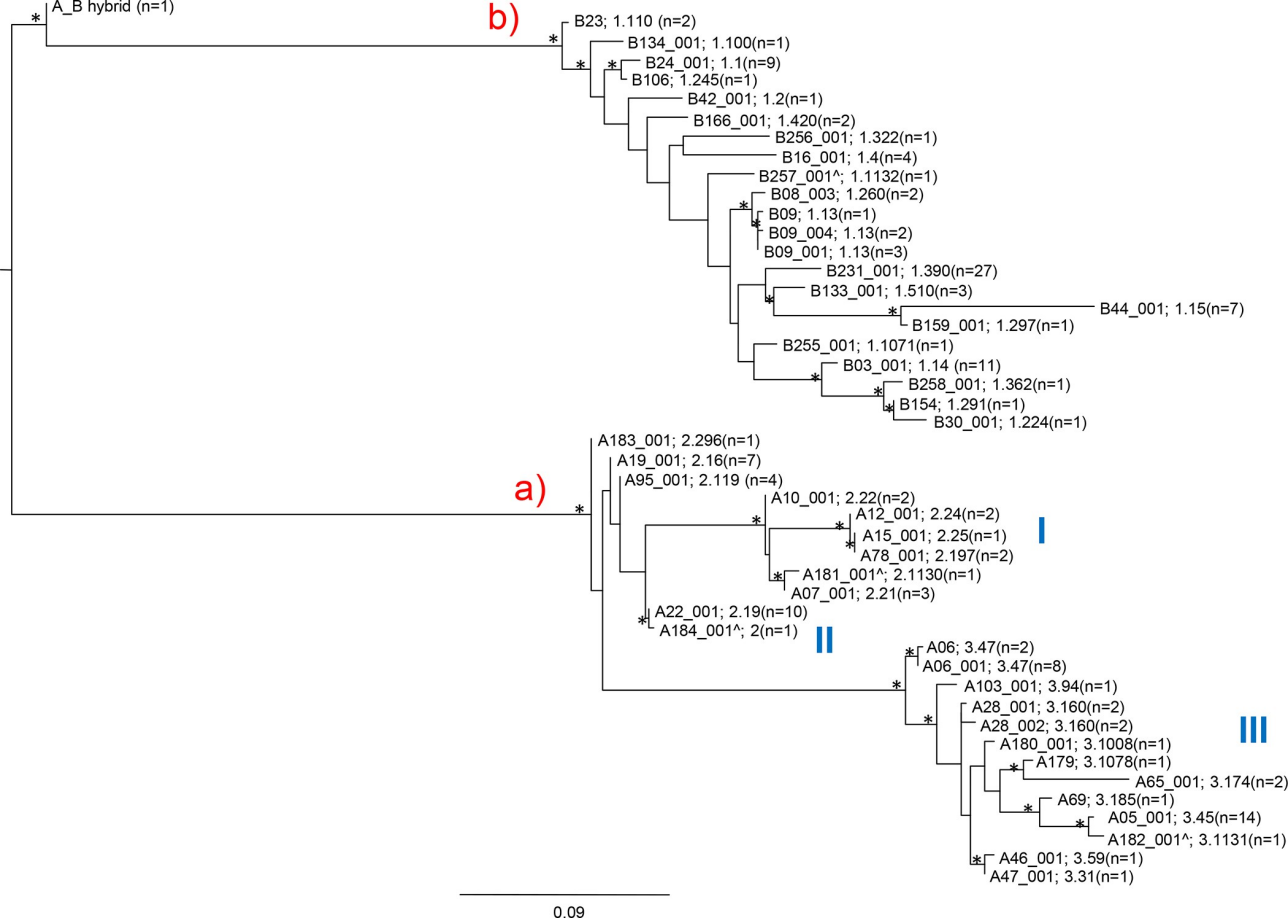
Maximum likelihood (ML) phylogenetic tree computed on *Neisseria meningitidis* serogroup B human factor H binding protein (*fHbp*) nucleotide sequences. The tree was carried out using the GTR + I + G as the best evolutionary model. Branch lengths were drawn to scale with the bar at the bottom indicating 0.09 nucleotide substitutions per site. One asterisk along a branch represents significant statistical support for the clade subtending that branch (bootstrap values > 75%). The tree was midpoint rooted. Main clades and clusters were highlighted. Both vaccine nomenclatures were showed. The symbol ^ indicates the new variant found in the previous study [12]. The variant A184_001^ contained an internal stop codon.

**Fig 3:**
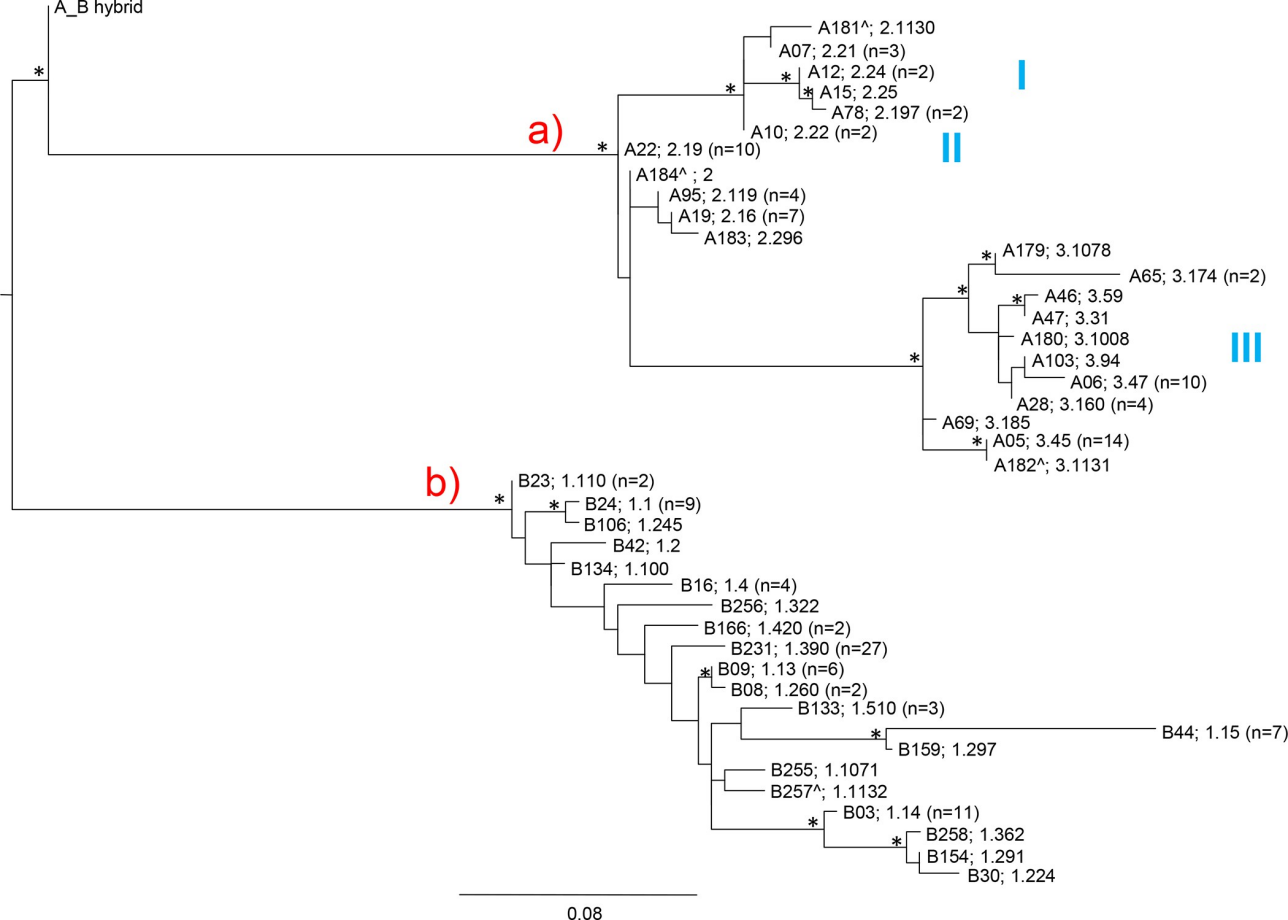
Maximum likelihood (ML) phylogenetic tree computed on *Neisseria meningitidis* serogroup B human factor H binding protein (FHbp) protein alignments. The tree was carried out using the JTT+ G as the best evolutionary model. Branch lengths were drawn to scale with the bar at the bottom indicating 0.08 nucleotide substitutions per site. One asterisk along a branch represents significant statistical support for the clade subtending that branch (bootstrap values >75%). The tree was midpoint rooted. Main clades and clusters were highlighted. Both vaccine nomenclatures were showed. The symbol ^ indicates the new variant found in the previous study [12]. The variant A184_001^ contained an internal stop codon.

Maximum likelihood (ML) phylogenetic trees were computed to provide a description of sequence relationships, to understand the relatedness of the FHbp variants, identified among MenB strains causing disease in Italy, from 2014 to 2017 (Figs 2 and 3).

The ML tree performed on *fHbp* nucleotide sequences (Fig 2) showed the two subfamilies A and B and the three variants (v1, v2 and v3) located into two main supported clades a) and b) (for subfamilies A and B, respectively). The sequence A_B hybrid appeared located outside the main clade b).

MenB clustered in the two clades, and their clusters, regardless to the year of isolation and patient’s age. Within the subfamily B (v1) clade many statistically supported clusters were outlined (Fig 2).

In particular, a close relationship was highlighted among the following FHbp variants (Fig 2):

- B24_001 (v1.1) and B106 (v.1.245);
- B08_003 (v.1.260) with B09 (v1.13), B09_004 (v. 1.13) and B09_001 (v. 1.13);
- B133_001 (v.1.510) with B44_001 (v.1.15) and B159_001 (v. 1.297);
- B03_001 (v.1.14) with B258_001 (v. 1.362), B154 (v.1.291) and B30_001 (v. 1.224).

The B257_001^ FHbp variant (v1.1132) was a new variant described in the previous study [13].

Within the subfamily A (v2 and v3) clade, three major clusters (I, II, III) were outlined. The cluster I included A10_001 (v2.22), A12_001 (v2.24), A15_001 (v2.25), A78_001 (v2.197), A07_001 (v2.21) and A181_001^ (v2.1130). The latter was a new variant [13] and related with A07_001 (v2.21). Cluster II included the new variant A184_001^ (v2) [13], related with A22_001 (v2.19). The sequence of A184_001^ (v2) contained an internal stop codon. Cluster III included a higher number of variants located in many supported clusters. The new variant A182_001^ (v3.1131) [13], belonging to cluster III, resulted related to A05_001 (v3.45), and A69 (v3.185) FHbp variants (Fig 2).

The ML tree performed on FHbp protein alignments showed the same topology, main clade a) and b) respectively for subfamilies A and B, and same internal clusters (except two sequences A22 (v2.19) and A184^ (2) which did not result located in a specific internal cluster) (Fig 3).

### Selective pressure analysis

Selective pressure analysis did not reveal any statistically supported positively selected sites (dN/dS > 1) on FHbp subfamily A sequences by using both HYPHY and Datamonkey REL method.

A total of 24 amino acid sites on the coding region of *fHbp* gene were identified by FEL method as negatively selected and statistically supported (Table 1 panel a).

**Table 1:** a) Selective pressure analysis on subfamily A fHBP gene sequences from *Neisseria meningitidis* of serogroup B strains. The reference sequence Factor H-binding protein (Uniprot_C6KHT4) has been used to trace the exact position of the amino acids found under selection. b) Selective pressure analysis on subfamily B fHBP gene sequences from *Neisseria meningitidis* of serogroup B strains. The reference sequence Factor H-binding protein (Uniprot_C6KHT4) has been used to trace the exact position of the amino acids found under selection.

A	
Positively selected sites (w for sites > 1) HYPHY	-
Negatively selected sites (w for sites < 1) HYPHY software	25 (G); 32 (G); 39 (L); 40 (T); 52 (S); 58 (S); 65 (L);
	67 (L); 83 (N); 87 (L); 97 (F); 102 (E); 109 (T); 149 (L);
	150 (V); 160 (F); 178 (D); 182 (G); 186 (Y); 190 (F);
	203 (K); 207 (Q); 239 (T); 249 (A)
B	
Positively selected sites (w for sites > 1) HYPHY and PAML software	28 (A; T); 33 (A; T); 49 (S; G); 57 (Q; D); 92 (V; I);
	106 (Q; K; R); 128 (L; F); 138 (H; D); 147 (Q; R);
	166 (G; V); 167 (M; S; R); 215 (D; Y); 222 (H; R);
	235 (A; D); 248 (Q; K); 265 (H; R; Q).
Negatively selected sites (w for sites < 1) HYPHY software	24 (G); 26 (G); 32 (G); 40 (T); 41 (A); 34 (L); 79 (G); 82 (L);
	91 (K); 94 (R); 95 (F); 99 (R); 104 (D); 112 (S); 126 (T);
	130 (T); 135 (D); 139 (S); 152 (D); 155 (G); 156 (E);
	174 (A); 180 (A); 181 (G); 186 (Y); 187 (T); 190 (F); 191 (A);
	193 (K); 205 (P); 209 (V); 211 (L); 217 (K); 233 (N); 236 (E);
	241 (S); 242 (L); 243 (G); 255 (S); 257 (E); 268 (G); 273 (Q).

Selective pressure analysis identified 16 amino acid sites on the coding region of *fHbp* among the subfamily B sequences, by REL—HYPHY and Datamonkey, as positively selected and statistically supported sites (dN/dS > 1), (Table 1 panel b). According to Uniprot Sequence feature description the amino acid residues from 120 to 273 (Uniprot Identifier: C6KHT4) correspond to the Lipoprot_C domain. Our results revealed that 10 of the 16 (62.5%) positively selected sites of the FHbp subfamily B sequences were located in this domain, showing the tendency of *N*. *meningitidis* serogroup B *fHbp* gene to vary in important regions. Meanwhile, only one positively selected site (57 Q; D) was located inside the regions of coiled coil within the protein. According to our results, two positive selected sites (248 and 265, positions related to Uniprot: C6KHT4) were located in the stretch of 27 residues, as previously reported (Malito et al., 2013) which also included the dodecapeptide C–terminal epitope. When considering the four discontinuous fragments, encompassing the residues distributed in both the N- and C-terminal domains of FHbp, our results showed that a total of seven of the 16 (43.75%) positively selected sites in the subfamily B, were located in these fragments region positions. Moreover, 42 statistically supported amino acid sites were identified by FEL method (both HYPHY and Datamonkey) as negatively selected (Table 1 panel b).

## Discussion

The FHbp is able to selectively bind the human factor H that is the key regulator of the alternative complement pathway, with important implications for meningococcal pathogenesis [12, 26].

The molecular analysis confirms that the MenB FHbp variants of the selected MenB strains were grouped in two clades, corresponding to the two subfamilies A and B, as previously reported [27].

Our phylogenetic investigation identified the various FHbp variants, intermixed in different supported clusters for clade a (subfamilies A) and for clade b (subfamilies B), indicating genetic relatedness of FHbp variants within each subfamily.

Within the subfamily A, we were able to characterize the phylogenetic relationships of the variants and to identify three major clusters (I, II, III).

The most common subfamily A variant, A05, belonged to the cluster III, while, the new variant A181 (v2.1130), grouped in the cluster I, related to variant A07 (v2.21) and externally located to the variants A78 (v2.197), A15 (v2.25), A12 (v2.24) and A10 (v2.22). In the cluster II we found the new variant A184 (v2) related with A22 (v2.19). Cluster III included a higher number of variants located in many supported clusters, including the new variant A182 (v3.1131).

The new B257 FHbp variant (v1.1132) belonged to the subfamily B and clustered in clade b, while the most frequent subfamily B variant, B231 did not belong to any statistically supported subgroup.

Methods that quantify the strength and type of natural selection by estimating the ratio of non-synonymous to synonymous substitution (*ω*) using phylogenetic codon-substitution models, have proven particularly useful, especially in the context of infectious diseases [28–34].

In our previous study we showed a number of variants slightly higher in subfamily A strains (22 variants) than subfamily B (20 variants) [13], among the samples here evaluated.

The phylogenetic analysis, the main focus of the present study, identified three clusters for subfamily A and four for subfamily B, confirming the genetic diversity within serogroup B meningococci as previously reported [13].

Our study suggested that subfamily B FHbp dataset undergo to a positive selective pressure respect to subfamily A. Generally, non-synonomous substitutions are promoted under positive selection as is the case of our subfamily B dataset, in which some changes may have become fixed, probably indicating their important functional / biological role.

These data are also in agreement with a previous study [27] which reports that evolution operates under different constraints within each subfamily, with subfamily B exhibiting more mutation feasible overall [27]. Some of the positively selected sites here identified appeared located inside the Lipoprotein C (Lipoprot_C) domain or in the coiled coil of the protein, showing the tendency of *N*. *meningitidis* serogroup B *fHbp* gene to vary in these important regions.

Several previous epitope mapping studies have been performed on FHbp of *N*. *meningitidis* [26, 35–37]. Overall, these studies identified critical residues for specific binding by anti-var1(subfamily B), -2 (subfamily A), and -3 (subfamily A) antibodies, suggesting that many epitopes are located within regions of protein diversity, consistent with diversifying selection and in line with our data.

In particular, the study by Malito *et al*. [35] investigated the molecular basis for the binding of a bactericidal mAb and mAb 12C1 to FHbp and a C-Terminal Peptide Epitope (dodecapeptide) recognized by the mAb 12C1.

In accordance with our results, two positive selected sites were located in the stretch of 27 residue reported by Malito *et al*., [38] which also include the dodecapeptide C–terminal epitope. Considering the four discontinuous fragments (L34-L48, I89-F96, T107-137 and Y214-L251) reported by Malito *et al*., [38], 6 out of 16 (37.5%) positively selected sites (subfamily B), were located within these portions.

Our data underlines the sequence diversity of FHbp on the investigated meningococcal strains and the importance to monitor the variations and selective pressure on this protein.

Monitoring the genetic diversity and molecular evolution of the FHBP gene is also important within the routine meningococcal surveillance.

## Supporting information

S1 Table The 109 MenB ID genomes submitted in the PubMLST.org database and used to analyze FHbp subfamily A and B variants. (DOCX)
