# Supplementary material for: FHbp variants among meningococci of serogroup B in Italy: Evolution and selective pressure, 2014–2017
Source: PLoS One. 2023 Feb 16;18(2):e0277976. doi: 10.1371/journal.pone.0277976 (PMC9934395; doi:10.1371/journal.pone.0277976)
Supplement: S1 Table — (DOCX) [file pone.0277976.s001.docx]

**Table S1 The 109 MenB ID genomes submitted in the PubMLST.org database and used to analyze FHbp subfamily A and B variants**

| **BIGS_ID** | **COUNTRY** | **CONTINENT** | **YEAR** |
| --- | --- | --- | --- |
| 84044 | Italy | Europe | 2014 |
| 52810 | Italy | Europe | 2014 |
| 40334 | Italy | Europe | 2014 |
| 52811 | Italy | Europe | 2014 |
| 40336 | Italy | Europe | 2014 |
| 84045 | Italy | Europe | 2014 |
| 40337 | Italy | Europe | 2014 |
| 40338 | Italy | Europe | 2014 |
| 84046 | Italy | Europe | 2014 |
| 40339 | Italy | Europe | 2014 |
| 36193 | Italy | Europe | 2014 |
| 52814 | Italy | Europe | 2014 |
| 40325 | Italy | Europe | 2014 |
| 40340 | Italy | Europe | 2014 |
| 36194 | Italy | Europe | 2014 |
| 35100 | Italy | Europe | 2014 |
| 40327 | Italy | Europe | 2014 |
| 40341 | Italy | Europe | 2014 |
| 40342 | Italy | Europe | 2014 |
| 2623 | Italy | Europe | 2014 |
| 52815 | Italy | Europe | 2014 |
| 84047 | Italy | Europe | 2014 |
| 52823 | Italy | Europe | 2014 |
| 40348 | Italy | Europe | 2014 |
| 52825 | Italy | Europe | 2014 |
| 40344 | Italy | Europe | 2015 |
| 52816 | Italy | Europe | 2015 |
| 40329 | Italy | Europe | 2015 |
| 40345 | Italy | Europe | 2015 |
| 52821 | Italy | Europe | 2015 |
| 84048 | Italy | Europe | 2015 |
| 84049 | Italy | Europe | 2015 |
| 84050 | Italy | Europe | 2015 |
| 52925 | Italy | Europe | 2015 |
| 40349 | Italy | Europe | 2015 |
| 40350 | Italy | Europe | 2015 |
| 40352 | Italy | Europe | 2015 |
| 97856 | Italy | Europe | 2015 |
| 40354 | Italy | Europe | 2015 |
| 84051 | Italy | Europe | 2015 |
| 40331 | Italy | Europe | 2015 |
| 40357 | Italy | Europe | 2015 |
| 40360 | Italy | Europe | 2015 |
| 40332 | Italy | Europe | 2015 |
| 84054 | Italy | Europe | 2015 |
| 84055 | Italy | Europe | 2015 |
| 41643 | Italy | Europe | 2015 |
| 41645 | Italy | Europe | 2015 |
| 41662 | Italy | Europe | 2015 |
| 40358 | Italy | Europe | 2015 |
| 41655 | Italy | Europe | 2016 |
| 41657 | Italy | Europe | 2016 |
| 41658 | Italy | Europe | 2016 |
| 41663 | Italy | Europe | 2016 |
| 41664 | Italy | Europe | 2016 |
| 97866 | Italy | Europe | 2016 |
| 84058 | Italy | Europe | 2016 |
| 52931 | Italy | Europe | 2016 |
| 97867 | Italy | Europe | 2016 |
| 84059 | Italy | Europe | 2016 |
| 52937 | Italy | Europe | 2016 |
| 84060 | Italy | Europe | 2016 |
| 52938 | Italy | Europe | 2016 |
| 84061 | Italy | Europe | 2016 |
| 84062 | Italy | Europe | 2016 |
| 52939 | Italy | Europe | 2016 |
| 52940 | Italy | Europe | 2016 |
| 52941 | Italy | Europe | 2016 |
| 52942 | Italy | Europe | 2016 |
| 52943 | Italy | Europe | 2016 |
| 52944 | Italy | Europe | 2016 |
| 84067 | Italy | Europe | 2016 |
| 52945 | Italy | Europe | 2016 |
| 52946 | Italy | Europe | 2016 |
| 84069 | Italy | Europe | 2016 |
| 52948 | Italy | Europe | 2016 |
| 52949 | Italy | Europe | 2016 |
| 52950 | Italy | Europe | 2016 |
| 84070 | Italy | Europe | 2016 |
| 52953 | Italy | Europe | 2016 |
| 52954 | Italy | Europe | 2016 |
| 52957 | Italy | Europe | 2016 |
| 52958 | Italy | Europe | 2016 |
| 52960 | Italy | Europe | 2016 |
| 84074 | Italy | Europe | 2017 |
| 59077 | Italy | Europe | 2017 |
| 97855 | Italy | Europe | 2017 |
| 59080 | Italy | Europe | 2017 |
| 84081 | Italy | Europe | 2017 |
| 84083 | Italy | Europe | 2017 |
| 59079 | Italy | Europe | 2017 |
| 53716 | Italy | Europe | 2017 |
| 59083 | Italy | Europe | 2017 |
| 84086 | Italy | Europe | 2017 |
| 84087 | Italy | Europe | 2017 |
| 84088 | Italy | Europe | 2017 |
| 84089 | Italy | Europe | 2017 |
| 84090 | Italy | Europe | 2017 |
| 84091 | Italy | Europe | 2017 |
| 84092 | Italy | Europe | 2017 |
| 59078 | Italy | Europe | 2017 |
| 59082 | Italy | Europe | 2017 |
| 59081 | Italy | Europe | 2017 |
| 76940 | Italy | Europe | 2017 |
| 71800 | Italy | Europe | 2017 |
| 84098 | Italy | Europe | 2017 |
| 84102 | Italy | Europe | 2017 |
| 84103 | Italy | Europe | 2017 |
| 84107 | Italy | Europe | 2017 |
